# Using Remimazolam to Generate Hemodynamically Stable Burst Suppression: A Case Report and Literature Review

**DOI:** 10.1155/cria/7512576

**Published:** 2025-07-30

**Authors:** Chin Fung Kelvin Kan, Jacob E. Pollard

**Affiliations:** ^1^Department of Anesthesiology, University of Utah School of Medicine, Salt Lake City, Utah, USA; ^2^Department of Anesthesiology, Hospitals/Lincoln-NYC Health, New York City, New York, USA

## Abstract

Remimazolam is a short-acting benzodiazepine that was approved by the United States Food and Drug Administration (FDA) in 2020 for the induction and maintenance of procedural sedation in adults undergoing procedures lasting 30 min or less. Given its recent introduction, the use of remimazolam for general anesthesia and monitored anesthesia care (MAC) remains an area of ongoing investigation. In this report, we present the first documented case demonstrating that remimazolam can achieve hemodynamic stable burst suppression in a critically ill patient undergoing emergent craniectomy and aneurysm clipping. Additionally, this manuscript reviews the reported off-label applications of remimazolam in both the operating room and the intensive care unit (ICU) settings.

## 1. Introduction

Remimazolam is a short-acting benzodiazepine that was approved by the Food and Drug Administration (FDA) in 2020. It is currently indicated for the induction and maintenance of procedural sedation in adults undergoing procedures lasting 30 min or less [1]. Despite its limited approved use, emerging literature has described off-label applications of remimazolam in general anesthesia, highlighting benefits such as preserved spontaneous ventilation and enhanced hemodynamic stability. This report presents the first documented case in which remimazolam was successfully used to achieve burst suppression. Furthermore, we review current and potential applications of remimazolam within the field of anesthesiology. The case reported is presented in compliance with Health Insurance Portability and Accountability Act (HIPAA) privacy regulations.

## 2. Case Presentation

A 64-year-old female with an unknown medical history acutely developed nausea and a headache, after which she was found unresponsive on the ground. Her Glasgow Coma Scale (GCS) score was 5, promoting intubation at the scene prior to transport to an outside hospital (OSH). At the OSH, imaging revealed both subdural and subarachnoid hemorrhage. Given the need for a higher level of care, she was transferred to the Neurocritical Care Unit (NCCU) at the University of Utah Hospitals. However, during transport, her hemodynamic and neurological status rapidly deteriorated, necessitating diversion to the emergency department for stabilization. On arrival, she was hypotensive and acidotic. Management included initiation of 3% hypertonic saline and vasopressors support with vasopressin and norepinephrine infusions.

**Figure 1:**
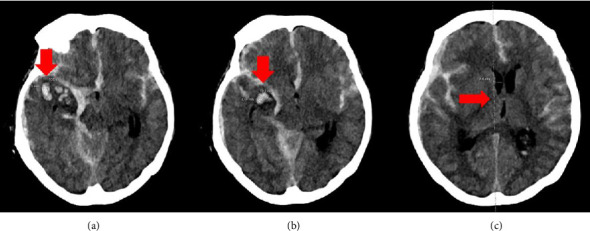
Subarachnoid hemorrhage and intraventricular hemorrhage and leftward midline shift. (a, b) Head CT demonstrating diffuse subarachnoid hemorrhage and a 4 × 2 × 2 mm right posterior communicating artery aneurysm. (c) Head CT showing diffuse cerebral edema with a 9 mm leftward midline shift, effacement of the suprasellar cistern, and medialization of the right uncus, concerning for developing descending transtentorial herniation.

A repeat computed tomography (CT) scan revealed a ruptured 2 mm right posterior communicating artery (PCoA) aneurysm, associated with a right convexity acute subdural and subarachnoid hemorrhage. These findings were consistent with Hunt–Hess grade 4 and modified Fisher grade 4 (Figures 1(a) and 1(b)). The hematoma also resulted in a significant right-to-left midline shift (Figure 1(c)). Given the severity of the presentation, the neurosurgery (NSGY) team proceeded with emergent surgical intervention for aneurysm clipping and evacuation of the right subdural hematoma.

Given the need for neuromonitoring, total intravenous anesthesia (TIVA) was selected for maintenance of anesthesia. At the start of the procedure, the patient was maintained on infusions of propofol (50 mcg/kg/min), remifentanil (0.1 mcg/kg/min), vasopressin (0.03 units/min), and norepinephrine (initially 0.03 mcg/kg/min, later titrated to 0.05 mcg/kg/min) to sustain a mean arterial pressure above 65 mmHg. When the NSGY team requested burst suppression, the anesthesia team opted to administer remimazolam, given the patient's reliance on vasopressors to preserve cerebral perfusion. In this context, propofol—though commonly used to achieve burst suppression—poised a high risk of inducing significant hypotension.

**Figure 2:**
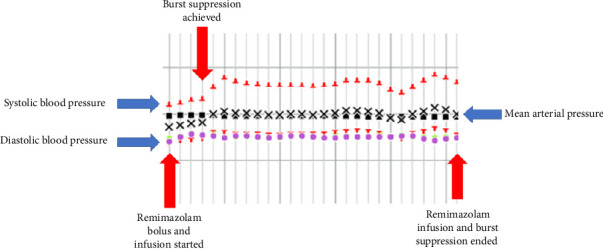
Intraoperative hemodynamics. Time course of key hemodynamic parameters during remimazolam-induced burst suppression. Systolic blood pressure (upper red triangles), diastolic blood pressure (lower red triangles), and mean arterial pressure (black Xs) are plotted over time. Each vertical line represents 1 min. Remimazolam bolus and infusion were initiated at the first red arrow. Burst suppression was achieved shortly thereafter and maintained during a dose reduction phase. At the final red arrow, the remimazolam infusion was discontinued, and burst suppression was terminated. Throughout the entire infusion period, blood pressures remained stable without the need for increased vasopressor support, demonstrating the hemodynamic stability of remimazolam in this setting.

A remimazolam infusion was initiated at 20 mg/h (0.25 mcg/kg/min) with a 5 mg bolus, resulting in the achievement of burst suppression within 4 min, as confirmed by electroencephalogram and the neuromonitoring technician. After 10 min, the infusion was reduced to 10 mg/hour while maintaining effective burst suppression. After 14 min, upon request from the NSGY team, burst suppression was discontinued and the remimazolam infusion was stopped. Throughout its administration, the patient remained hemodynamically stable without the need for increased vasopressor support (Figure 2), and the infusion rates of propofol and remifentanil were not adjusted. The remainder of the surgery proceeded uneventfully. Postoperatively, the patient was transferred to the NCCU while intubated, continuing on vasopressin and norepinephrine for hemodynamic support and maintained on propofol and fentanyl for sedation.

On postoperative day (POD) 1, the patient was successfully weaned off vasopressin and norepinephrine infusions. A cerebral angiogram demonstrated no residual aneurysm filling and no evidence of vasospasm. She was extubated on POD 11 and discharged to an acute inpatient rehabilitation facility on POD 26.

## 3. Discussion

Remimazolam, similar to midazolam and other benzodiazepines, enhances γ-aminobutyric acid type A (GABAA) receptor activity, resulting in neuron hyperpolarization through increased chloride ion influx and subsequent suppressing of neuronal signal transmission. Its effects can be rapidly reversed by flumazenil [2]. Remimazolam is classified as a “soft drug” due to its favorable pharmacokinetic profile including high clearance, low steady-state volume of distribution, predictable context-sensitive half-life, and metabolism by nonspecific tissue esterases—primarily hepatic carboxylesterase. It exhibits a rapid onset of action within 1 to 3 min and has a short terminal half-life of approximately 7 to 8 min [1].

**Figure 3:**
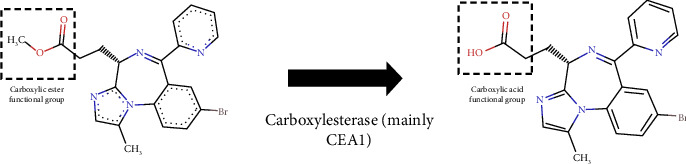
Remimazolam structure. Metabolism of remimazolam by carboxylesterase 1A (CES1A). On the left, the parent compound contains a carboxylic ester functional group (highlighted in the dashed box, red) that undergoes hydrolysis. Carboxylesterase (mainly CES1A) converts this ester into a carboxylic acid group (highlighted on the right), producing the inactive metabolite CNS705412. This transformation contributes to remimazolam's rapid metabolism and short duration of action [1].

Remimazolam's inactive carboxylic acid metabolite, CNS705412, exhibits approximately 300-fold lower affinity for GABAA receptors compared to the parent drug and is primarily excreted in the urine. As illustrated in Figure 3, remimazolam contains a carboxylic ester moiety, which is metabolized into a carboxylic acid mainly by carboxylesterase 1A (CES1A) [1]. This metabolic pathway is analogous to that of esmolol and remifentanil, both of which also possess ester linkages and undergo rapid metabolism via nonspecific tissue esterases.

Remimazolam's favorable hemodynamic profile has been documented in both monitored anesthesia care (MAC) and general anesthesia settings. For instance, Kotani et al. reported that patients undergoing transcatheter aortic valve replacement experienced higher arterial hypotension rates during induction with propofol-based TIVA compared to remimazolam-based TIVA [3]. A multicenter phase III trial in patients undergoing colonoscopy also demonstrated a lower rate of hypotension with remimazolam sedation relative to propofol. Additionally, Ko et al. found that remimazolam was associated with reduced hypotension compared to sevoflurane-based general anesthesia during cerebral aneurysm coil embolization [4]. Similarly, Zhang et al. showed that general anesthesia with remimazolam, compared to propofol, resulted in a lower incidence of intraoperative hypotension in patients undergoing cerebral endovascular procedures, although it did not significantly reduce the rates of postoperative delirium rate or emergence time [5].

In addition to its hemodynamic stability, studies have shown that remimazolam is associated with less respiration depression compared to propofol during MAC. While remimazolam offers several clinical advantages, there have been case reports of adverse events, including remimazolam-induced anaphylaxis, hemodynamic collapse, and episodes of hypotension [6, 7]. Therefore, further research is necessary to better define the incidence and risk factors associated with these complications.

In NSGY, where TIVA with a combination of remifentanil and propofol is commonly employed, remimazolam may serve as a suitable alternative to propofol. This potential substitution is supported by evidence suggesting synergistic effects between remimazolam and remifentanil [8]. Consequently, remimazolam may be an ideal agent for TIVA when paired with remifentanil. Importantly, remimazolam has been shown not to interfere with neuromonitoring [9]. Additionally, its use may help mitigate remimazolam-associated side effects such as opioid-induced hyperalgesia and chest wall rigidity. Huang et al. demonstrated that remimazolam-alfentanil TIVA regimen could safely and effectively replace propofol-alfentanil TIVA for maintaining general anesthesia in women undergoing hysteroscopic examination [10]. Nevertheless, further studies are warranted to evaluate whether remimazolam infusions can lead to acute tolerance or withdrawal.

Interestingly, both in vitro and in vivo studies have demonstrated that remimazolam exerts anti-inflammation effects through multiple pathways [11]. A recent study by Tsukimoto et al. further revealed that remimazolam possesses antioxidant properties, resulting in a more potent anti-inflammatory effect than dexmedetomidine [12]. These findings suggest a potential role for remimazolam in mitigating surgical-induced inflammation, which may be particularly beneficial in oncological surgeries. Additionally, its anti-inflammatory action may contribute to the observed reduction in postoperative nausea and vomiting [13]. However, while promising, this anti-inflammation effect has not yet been shown to reduce short-term surgical morbidity, and no current studies have demonstrated long-term mortality or morbidity benefits. Ongoing studies—such as the study by Kim et al. evaluating the effects of remimazolam versus propofol anesthesia on postoperative delirium in neurovascular surgery patients—may provide further insights into whether remimazolam's anti-inflammatory properties translate into meaningful clinical benefits [14].

Studies have shown that remimazolam does not require dosing adjustments in patients with renal or hepatic insufficiency [15]. However, optimal dosing in specific populations such as the obese and elderly remains unclear. Additionally, patients with a history of chronic benzodiazepine use may exhibit tolerance to remimazolam, complicating dose titration in this group. Beyond these special populations, the fact that remimazolam is primarily metabolized by CES1 raises concerns about altered pharmacokinetics and pharmacodynamics in individuals with CES1 deficiency or genetic polymorphisms affecting enzyme activity.

Finally, remimazolam may serve as a valuable sedation agent in the intensive care unit (ICU) due to its rapid onset, hemodynamically stability, and short offset. Additionally, emergent evidence suggests that remimazolam does not increase the risk of perioperative neurocognitive disorders [16]. This raises the potential for future studies to explore whether prolonged infusions are associated with ICU delirium. Other potential applications for remimazolam infusion that we propose include the management of muscle spasm–induced pain, ICU-related anxiety, treatment or prevention of status epilepticus, and the management of delirium tremens and alcohol withdrawal.

This report presents the first documented case of using remimazolam to achieve hemodynamic stable burst suppression during emergent craniectomy and aneurysm clipping. Further studies are warranted to compare remimazolam-induced burst suppression with that of propofol, particularly in evaluating potential differences in surgical mortality and morbidity.

## Data Availability

The data that support the findings of this study are available on request from the corresponding author. The data are not publicly available due to privacy or ethical restrictions.
